# Supplementary material for: Drug‐induced increase in lysobisphosphatidic acid reduces the cholesterol overload in Niemann–Pick type C cells and mice
Source: EMBO Rep. 2019 May 22;20(7):e47055. doi: 10.15252/embr.201847055 (PMC6607015; doi:10.15252/embr.201847055)
Supplement: Supplementary file 1 — Appendix [file EMBR-20-e47055-s001.pdf]

Table of Content:

Appendix Figure S1. Page 3

Appendix Figure S2. Page 4

Appendix Figure S3. Page 5

Appendix Figure S4. Page 6

Appendix Figure S5. Page 7

Appendix Figure S1. LBPA analysis by mass spectrometry

(A) HILIC liquid chromatography separation of sLBPA, LBPA and PG standards. Extracted ion chromatograms of positive precursor ions at 2ppm mass tolerance of sLBPA (54:3) 1052.8202Da, (42:0) 894.6793Da; LBPA (28:0) 684.4810Da; and PG (28:0)684.4810Da, (28:2) 680.4497Da, (36:2) 792.5749Da

(B) Separation by endogenous LBPA, SLBPA, PG and PS detected in mouse liver extracts (y axis: LC elution time; x axis: m/z of the positive precursor ion. PG: phosphatidylglycerol; LBPA: lysobisphosphatidic acid; sLBPA: semi- lysobisphosphatidic acid; PS: phosphatidylserine.

Appendix Figure S2. Analysis of sLBPA in mouse liver.

A, B) *Npc1*$^{-/-}$and *Npc1*$^{+/+}$ mice were treated or not with thioperamide, and liver extracts were prepared. All sLBPA species present in the extracts were quantified by LC-MS (as in Fig EV5), and the sum of all species is expressed as a percentage of all phospholipids (A). From the experiments in (A). the total amount of major phospholipid classes in mouse liver are expressed as a percentage of all phospholipids (B). PG: phosphatidylglycerol; LBPA: lysobisphosphatidic acid; PS: phosphatidylserine; PI: phosphatidylinositol; PC: phosphatidylcholine; PE: phosphatidylethanolamine. n=3 independent experiments, error bars = SD.

Appendix Figure S3 Distribution of phosphatidylcholine (PC) and phosphatidylethanolamine (PE) species in mouse liver extracts

A, B) The experiment and analysis are as in Fig EV7, except that PC (A) and PE (B) species were quantified.

Appendix Figure S4 Behavioral analysis of thioperamide-treated mice

Thioperamide treatment alone did not significantly improve the life span, motor function/rearing or the high frequency tremor in Npc1-/- mice, however, some benefits were observed when combined with miglustat treatment (6 mice per condition).

A) Median survival for untreated Npc1-/- mice was 83 days, thioperamide (2mg/kg/day) treated Npc1-/- mice was 83 days, miglustat (600mg/kg) treated Npc1-/- mice was 139.5 days, and miglustat + thioperamide combination treated Npc1-/- mice was 146 days.

B) Average weekly body weight measurements.

C) No significant improvement in rearing ability was observed in the thioperamide treated mice, and no significant difference was observed between the miglustat and the miglustat/thioperamide combination treated group.

D) Thioperamide treatment increased high frequency tremor at 11 weeks of age compared to untreated mice. At 14 and 17 weeks, when all thioperamide and untreated mice had reached humane end point, mice treated with miglustat alone had a significantly elevated HF tremor compared to wild type mice. However, some improvement was observed with the combined miglustat/thioperamide treatment.

Appendix Figure S5 Distribution of cholesterol and LBPA in mouse brain extracts.

(A, B) *Npc1*$^{-/-}$and *Npc1*$^{+/+}$ mice were treated or not with thioperamide for 6 weeks after weaning and sacrificed (6 mice per condition). The total content of unesterified cholesterol (A) in the corresponding brain extracts was then quantified using an enzymatic assay and is expressed in nmol per mg tissue. The total content of LBPA (B) in the same liver extracts was quantified by mass spectrometry, and is therefore expressed as a percentage of total phospholipids, error bars = SD.

(C) The species of LBPA were quantified by LC-MS brain extracts of *Npc1*$^{-/-}$and *Npc1*$^{+/+}$ mice, treated or not with thioperamide, and are expressed as a percentage of the total. The color code is the same as in Fig EV7, and indicates the acyl chain composition of the various species listed in the captions, with the total number of carbon atoms of the 2 acyl chains together, and the degree of insaturation. The prefix “e” indicates lipids with one 1-O-alkyl bond; prefix “p” indicates lipids with one 1-O-alkenyl bond.

A

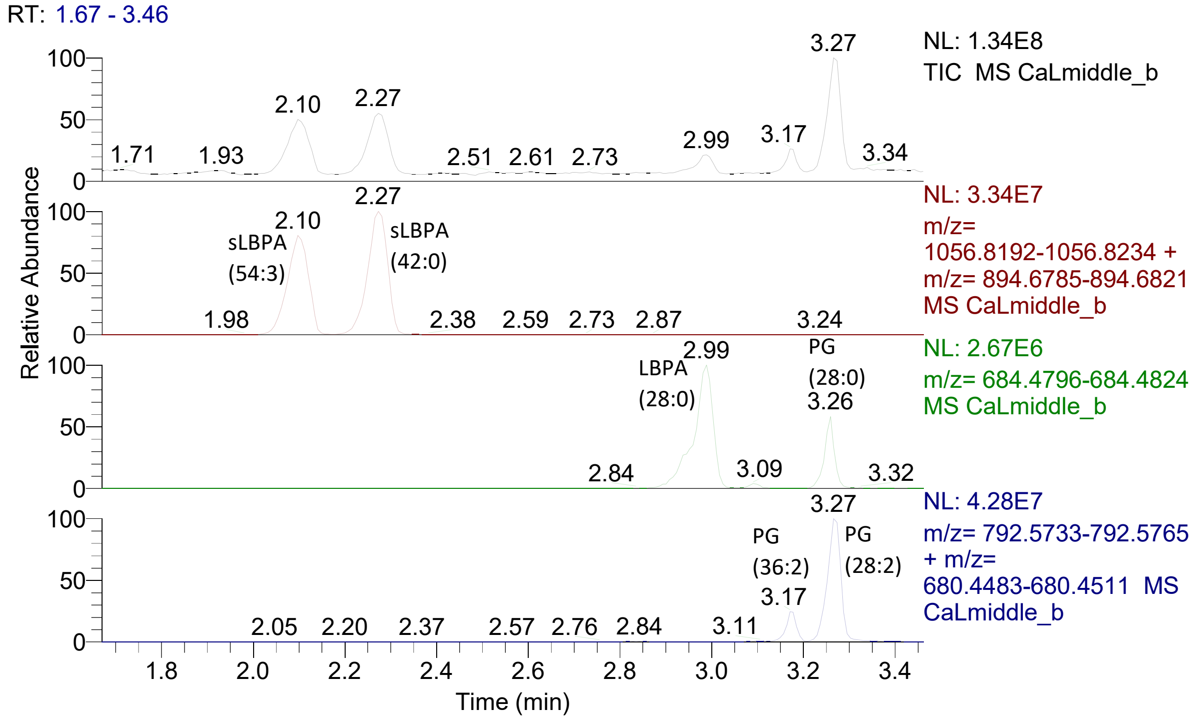
RT: 1.67 - 3.46
NL: 1.34E8
TIC MS CaLmiddle_b
NL: 3.34E7
m/z= 1056.8192-1056.8234 + m/z= 894.6785-894.6821 MS CaLmiddle_b
sLBPA (54:3)
sLBPA (42:0)
NL: 2.67E6
m/z= 684.4796-684.4824 MS CaLmiddle_b
LBPA (28:0)
PG (28:0)
NL: 4.28E7
m/z= 792.5733-792.5765 + m/z= 680.4483-680.4511 MS CaLmiddle_b
PG (36:2)
PG (28:2)
Relative Abundance
Time (min)

B

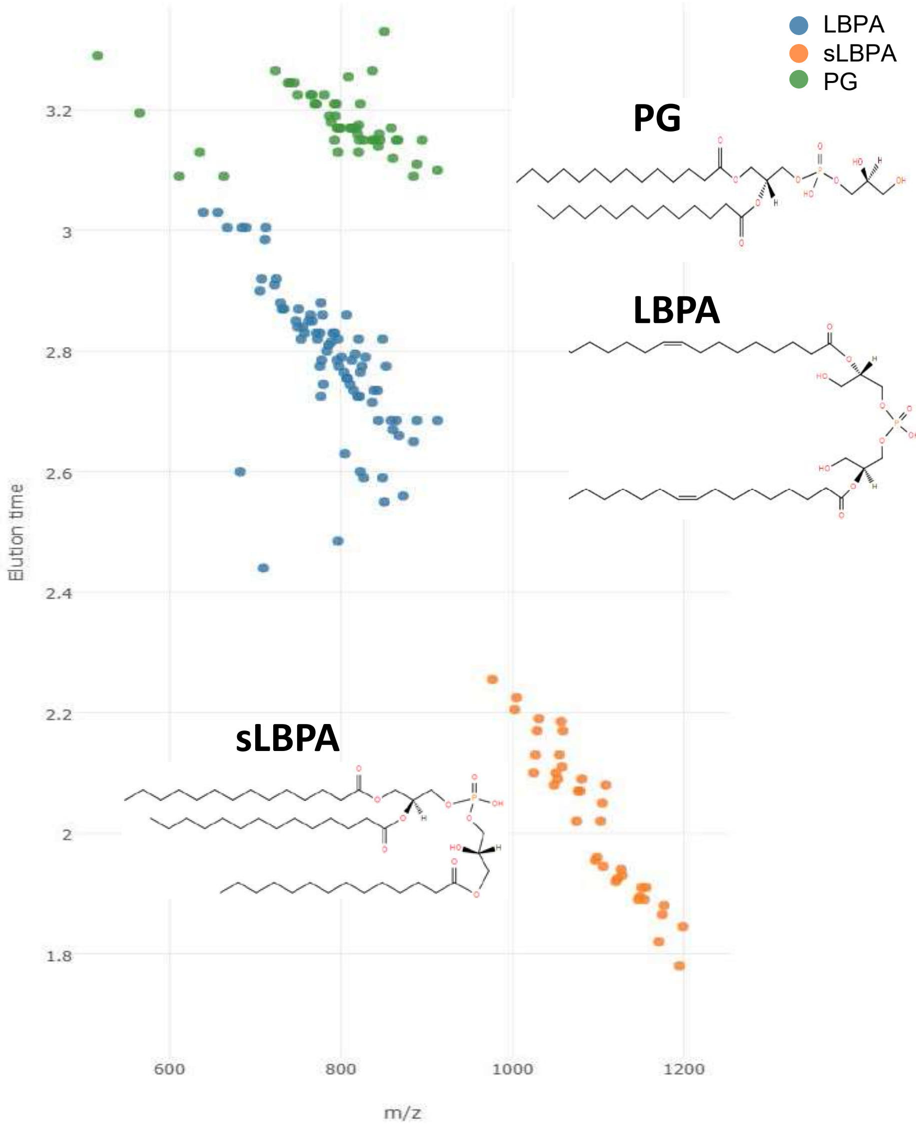
LBPA
sLBPA
PG
PG
LBPA
sLBPA
Elution time
m/z

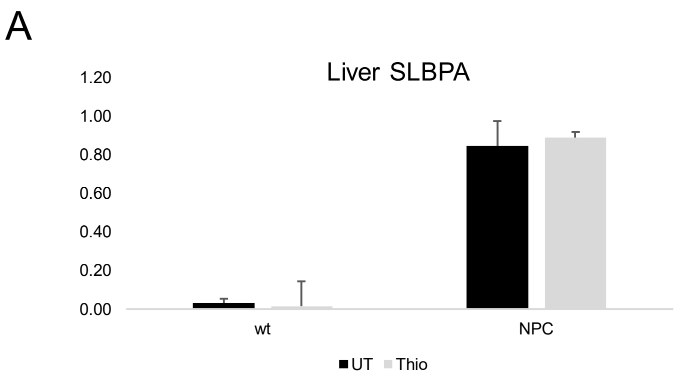
A
Liver SLBPA
1.20
1.00
0.80
0.60
0.40
0.20
0.00
wt
NPC
UT
Thio

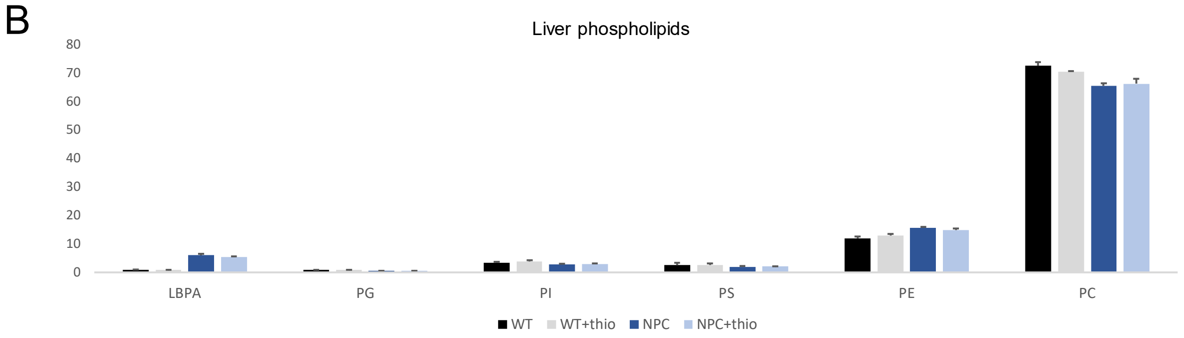
B
Liver phospholipids
80
70
60
50
40
30
20
10
0
LBPA
PG
PI
PS
PE
PC
WT
WT+thio
NPC
NPC+thio

A

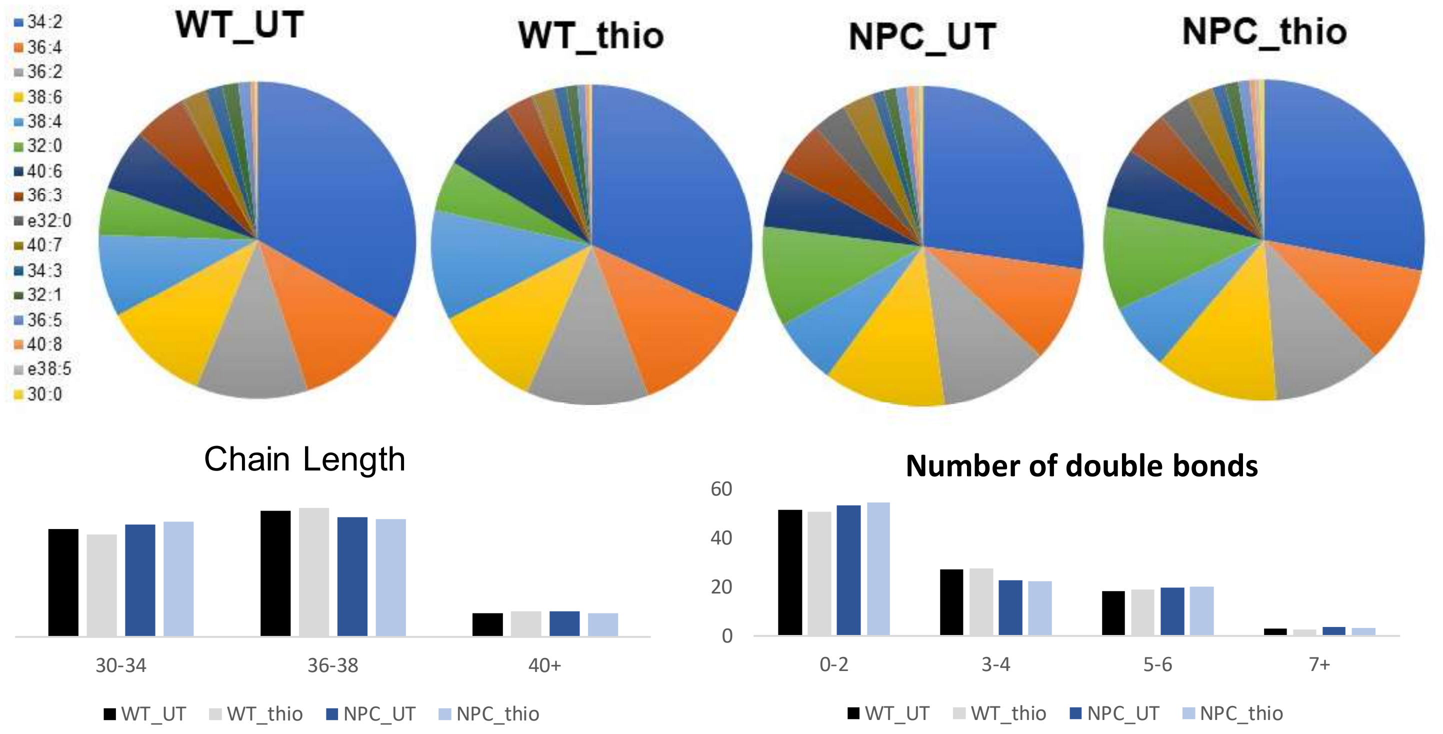
WT_UT
WT_thio
NPC_UT
NPC_thio
34:2
36:4
36:2
38:6
38:4
32:0
40:6
36:3
e32:0
40:7
34:3
32:1
36:5
40:8
e38:5
30:0
Chain Length
60
40
20
0
30-34
36-38
40+
WT_UT WT_thio NPC_UT NPC_thio
Number of double bonds
60
40
20
0
0-2
3-4
5-6
7+
WT_UT WT_thio NPC_UT NPC_thio

B

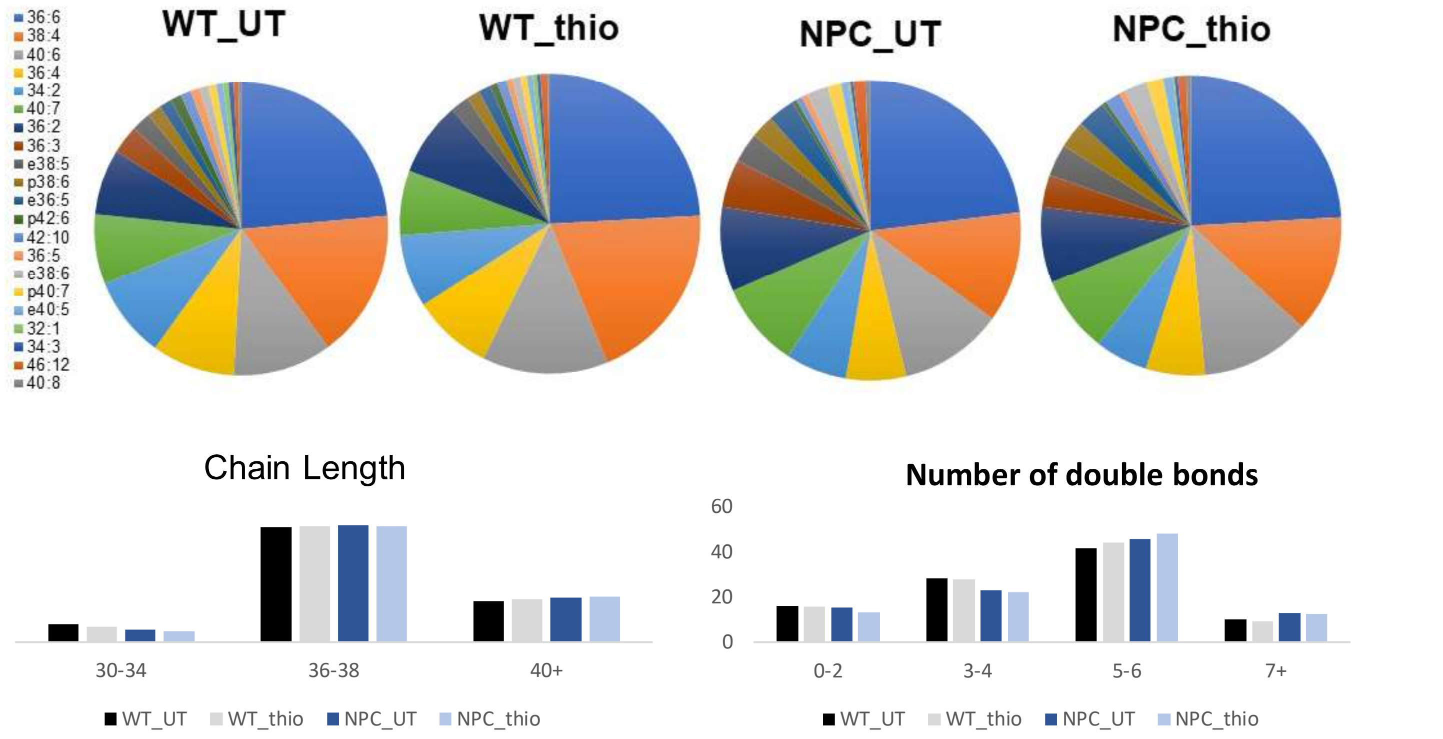
WT_UT
WT_thio
NPC_UT
NPC_thio
36:6
38:4
40:6
36:4
34:2
40:7
36:2
36:3
e38:5
p38:6
e36:5
p42:6
42:10
36:5
e38:6
p40:7
e40:5
32:1
34:3
46:12
40:8
Chain Length
80
60
40
20
0
30-34
36-38
40+
WT_UT WT_thio NPC_UT NPC_thio
Number of double bonds
60
40
20
0
0-2
3-4
5-6
7+
WT_UT WT_thio NPC_UT NPC_thio

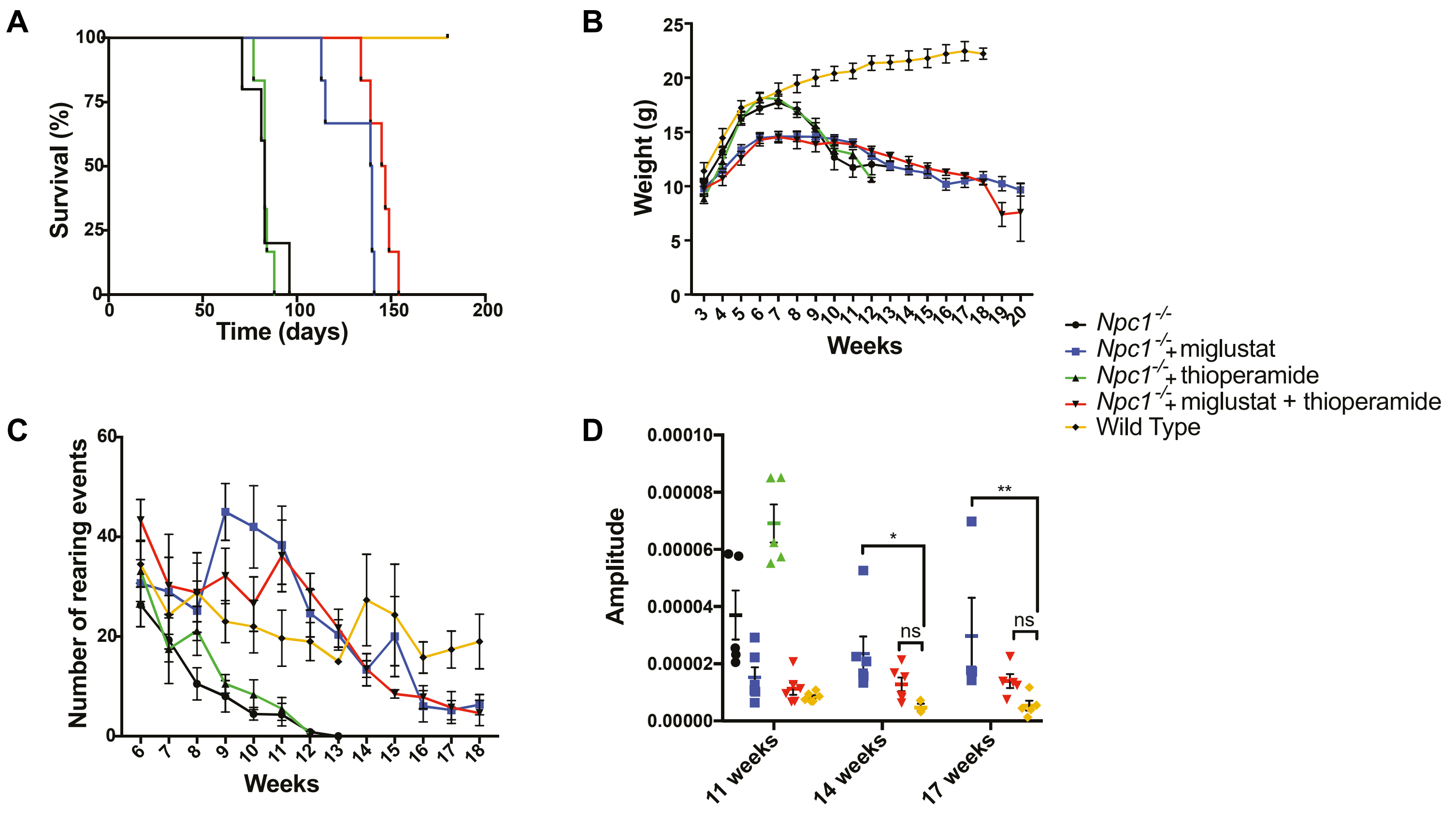
A
Survival (%)
Time (days)
B
Weight (g)
Weeks
C
Number of rearing events
Weeks
D
Amplitude
11 weeks
14 weeks
17 weeks
*
**
ns
ns
Npc1-/-
Npc1-/-+miglustat
Npc1-/-+thioperamide
Npc1-/-+miglustat + thioperamide
Wild Type

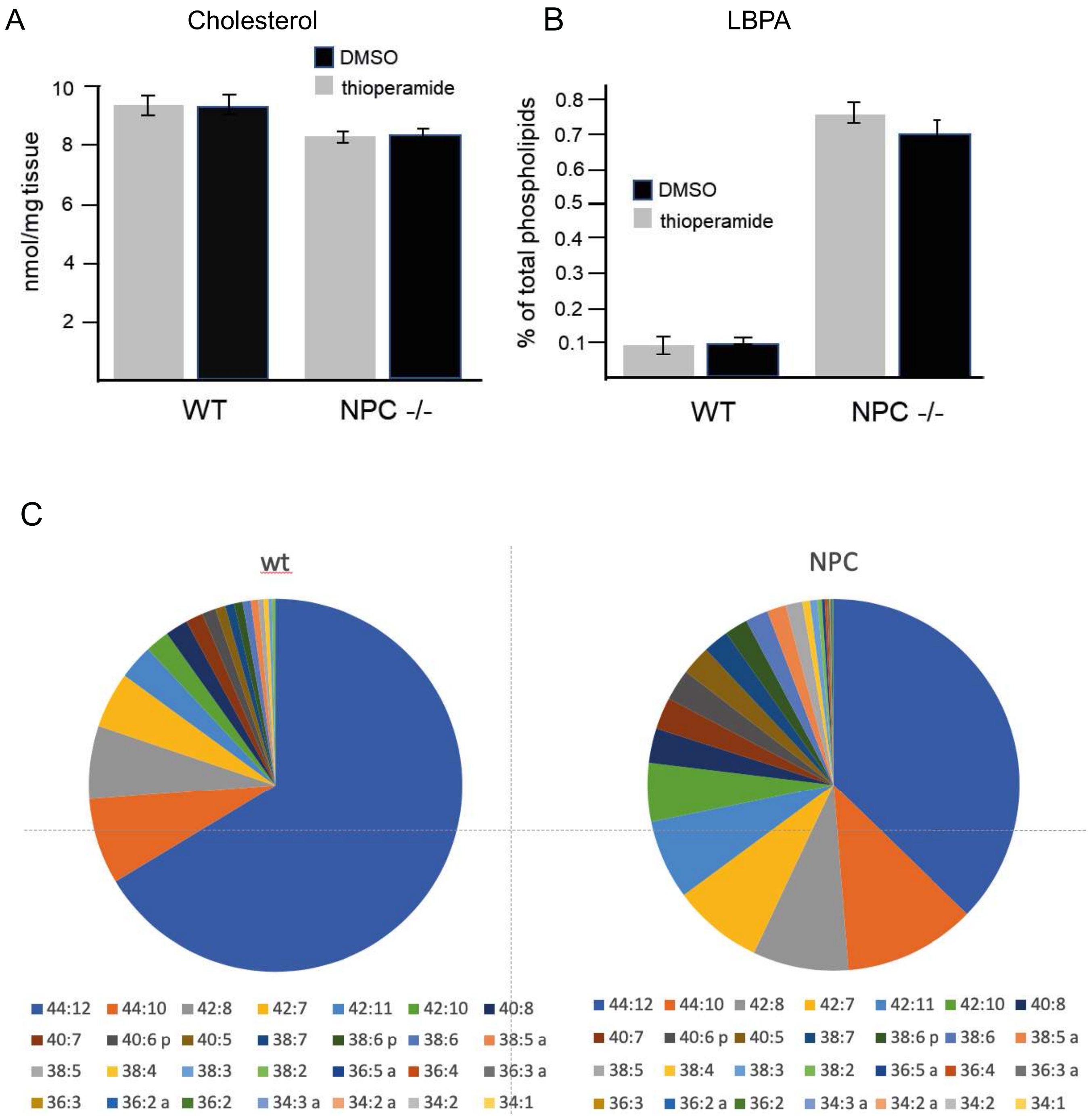
A
Cholesterol
DMSO
thioperamide
nmol/mg tissue
10
8
6
4
2
WT
NPC -/-
B
LBPA
% of total phospholipids
0.8
0.7
0.6
0.5
0.4
0.3
0.2
0.1
DMSO
thioperamide
WT
NPC -/-
C
wt
NPC
44:12 44:10 42:8 42:7 42:11 42:10 40:8
40:7 40:6 p 40:5 38:7 38:6 p 38:6 38:5 a
38:5 38:4 38:3 38:2 36:5 a 36:4 36:3 a
36:3 36:2 a 36:2 34:3 a 34:2 a 34:2 34:1
44:12 44:10 42:8 42:7 42:11 42:10 40:8
40:7 40:6 p 40:5 38:7 38:6 p 38:6 38:5 a
38:5 38:4 38:3 38:2 36:5 a 36:4 36:3 a
36:3 36:2 a 36:2 34:3 a 34:2 a 34:2 34:1
