# Supplementary material for: Drug‐induced increase in lysobisphosphatidic acid reduces the cholesterol overload in Niemann–Pick type C cells and mice
Source: EMBO Rep. 2019 May 22;20(7):e47055. doi: 10.15252/embr.201847055 (PMC6607015; doi:10.15252/embr.201847055)
Supplement: Supplementary file 2 — Expanded View Figures PDF [file EMBR-20-e47055-s002.pdf]

Expanded View Figures

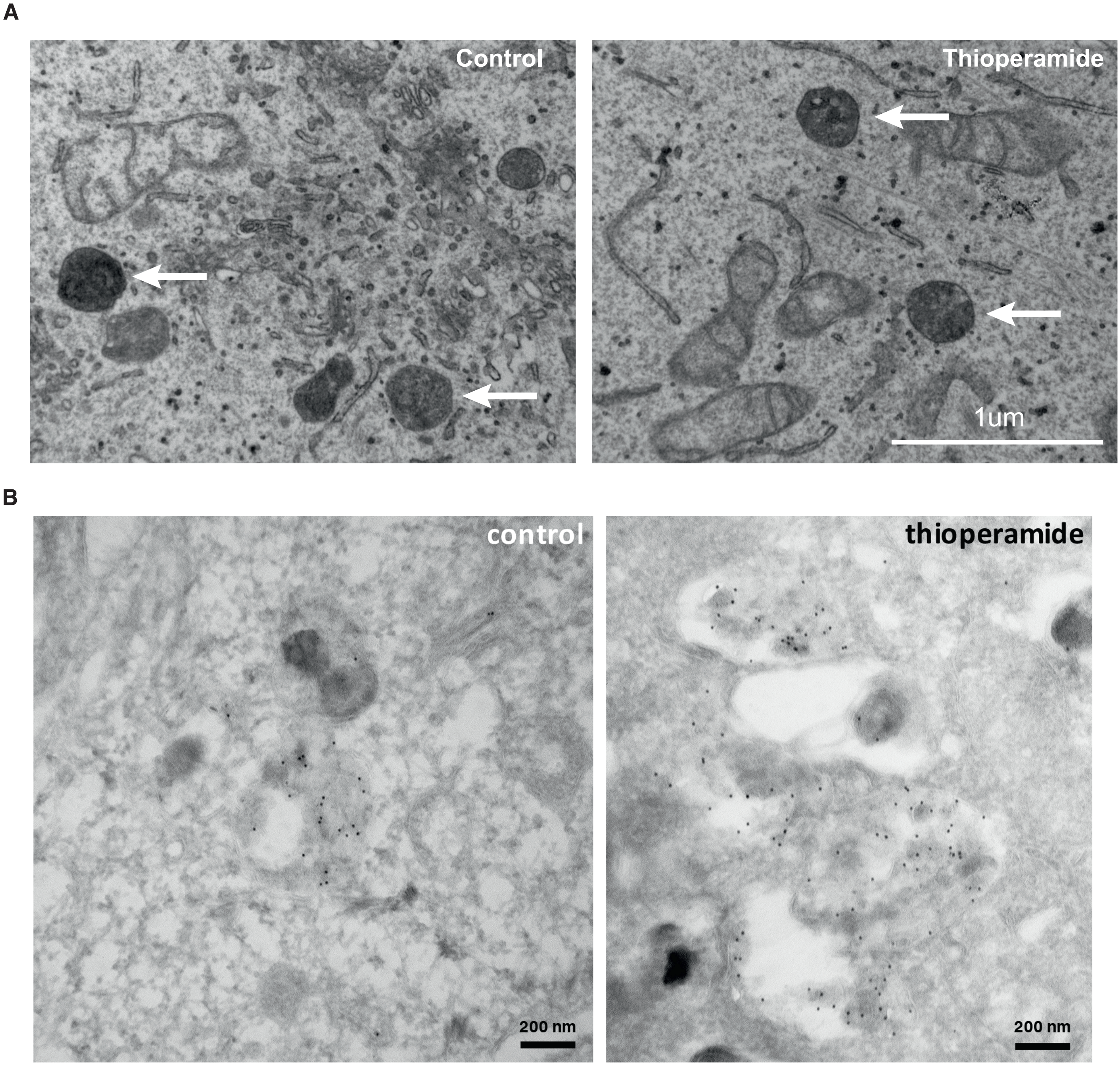

Figure EV1. Electron microscopy analysis of thioperamide-treated cells.

A, B HeLa MZ cells were treated or not with thioperamide, embedded in Epon and processed for electron microscopy (A). Arrows point at structures with the characteristic appearance of multivesicular endosomes. Alternatively, cryosections were prepared and labelled with anti-LBPA antibodies followed by 5 nm protein A-gold (B); micrographs are same as in Fig 3A, but without pseudo-colouring. Scale bars: (A) 1 μm; (B) 100 nm.

 © 2019 The Authors

Figure EV2. Thioperamide does not alter endosomal functions.

A Analysis of endosomal compartments by immunofluorescence. HeLa MZ cells were treated or not with thioperamide or U18666A for 18 h and processed for immunofluorescence microscopy after labelling with DAPI (nuclei) and antibodies against the indicated proteins. Lipid droplets were labelled with bodipy [14]. The bar graph shows the quantification by automated microscopy of the intensity of the fluorescence signals; TfR: transferrin receptor (n = 3 independent experiments, 200 images analysed per experiments, error bars = SD).

B Infection with vesicular stomatitis virus (VSV). HeLa cells treated or not with thioperamide for 15 h were further incubated for 3 h in the presence of the drug and recombinant VSV expressing a GFP-tagged version of the viral phosphoprotein (P-eGFP) [22,23] at low physiologically relevant MOI (1.0). Cells were labelled with DAPI (nuclei) and analysed by fluorescence microscopy. The bar graph shows the percentage of infected cells after quantification by automated microscopy (n = 3 independent experiments, 100 images analysed per experiments, error bars = SD, two-way ANOVA, ****$P < 0.0001$).

C Acidic pH of endosomes and lysosomes. HeLa cells were treated or not with thioperamide as in (A) and incubated with LysoTracker to label acidic compartments. For comparison, cells were also treated for a short (2 h) period with the V-ATPase inhibitor bafilomycin A1. Cells were then labelled with DAPI (nuclei) and processed for fluorescence microscopy. The bar graphs show the integrated intensity (left) and number (right) of labelled structures after quantification by automated microscopy (n = 3 independent experiments, 64 images analysed per experiments, error bars = SD).

D EGF receptor degradation. HeLa MZ cells treated or not with thioperamide as in (A) were challenged with 100 nM EGF for 60 min and further incubated at 37°C for the indicated time periods with or without the drug, up to a total time period of 18 h. Cells were labelled with DAPI (nuclei) and antibodies against the EGF receptor and processed for fluorescence microscopy. The panels show examples of the EGF receptor distribution after 60 min. The bar graph shows the integrated intensity of the labelled structures after quantification by automated microscopy (n = 3 independent experiments, 100 images analysed per experiments, error bars = SD).

© 2019 The Authors

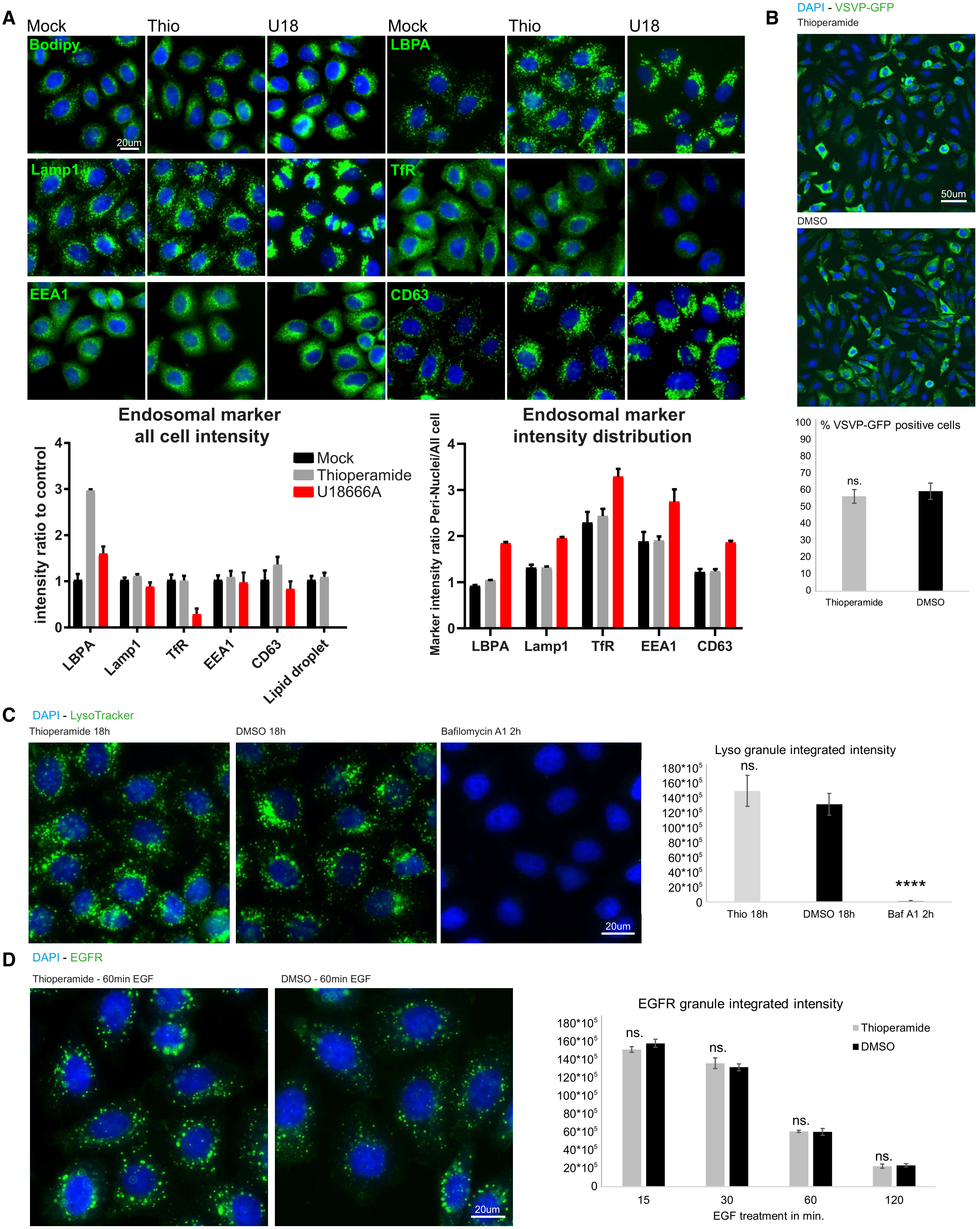

Figure EV2.

 © 2019 The Authors

Figure EV3. Dose–response and time course of thioperamide and pitolisant.

A HeLa MZ or A431 cells were treated with DMSO (0 control, black bars), thioperamide or pitolisant at the indicated concentrations for 18 h. Cells were labelled with anti-LBPA antibodies and analysed by automated microscopy. The intensity of LBPA staining was quantified and is normalized to the DMSO controls (*n* = 3 independent experiments with 288 images analysed per condition, error bars = SD).

B HeLa MZ cells were treated with DMSO, with 10 μM thioperamide or with 10 μM pitolisant for the indicated time periods. Cells were labelled with anti-LBPA antibodies and analysed by automated microscopy. The intensity of LBPA staining was quantified and is normalized to the DMSO controls (*n* = 3 independent experiments with 288 images analysed per condition).

C As in (B), nuclei were stained with DAPI and the number of nuclei per micrograph was quantified (*n* = 3 independent experiments with 288 images analysed per condition).

© 2019 The Authors

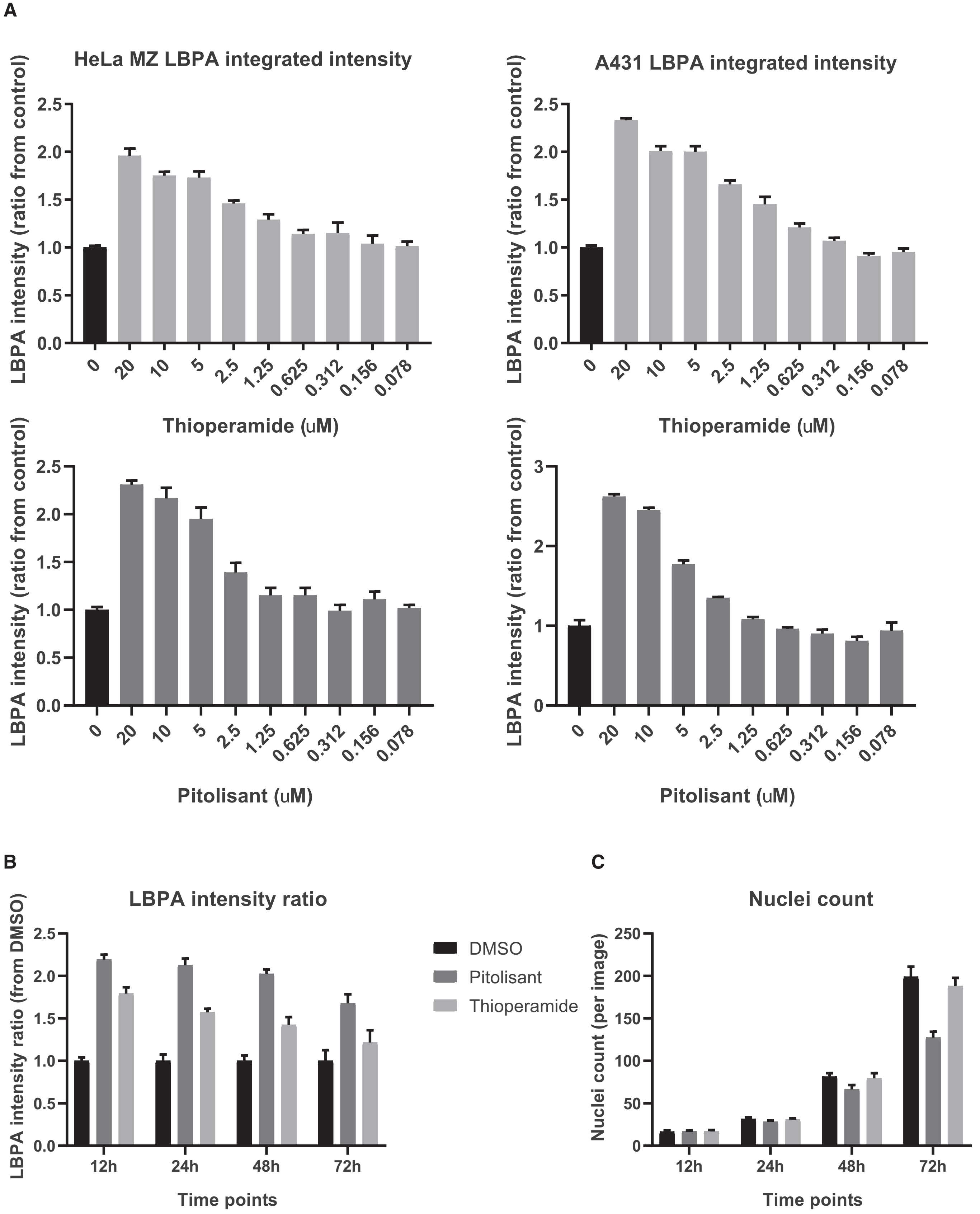

Figure EV3.

 © 2019 The Authors

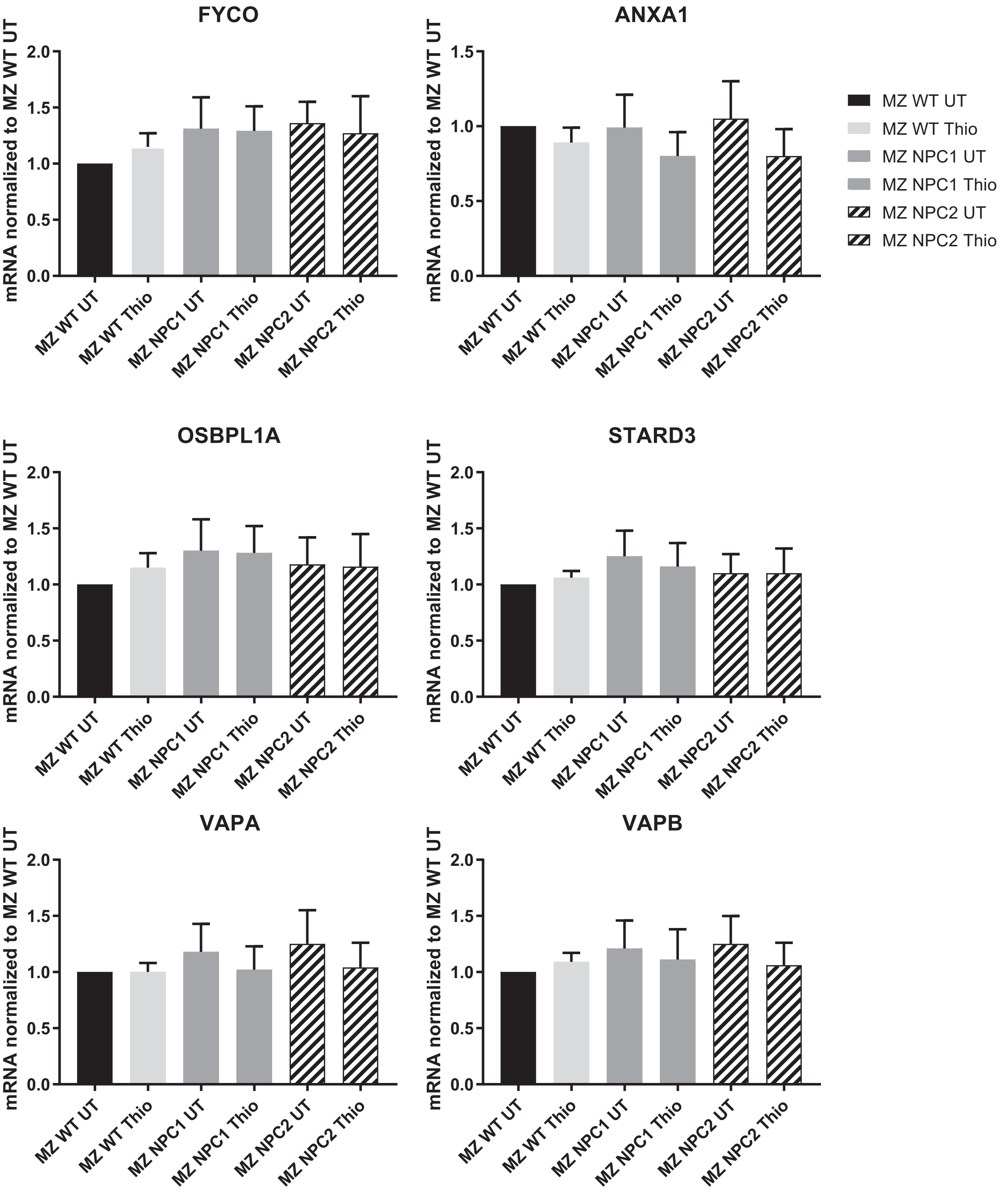

Figure EV4. Effects of thioperamide on the expression of proteins involved in membrane contact sites.
The parental HeLa MZ cells, NPC1 KO cells or NPC2 KO cells were treated or not with thioperamide for 18 h. Total mRNA was extracted, and the indicated mRNAs were quantified by RT–PCR (n = 3 independent experiments, error bars = SD).

© 2019 The Authors

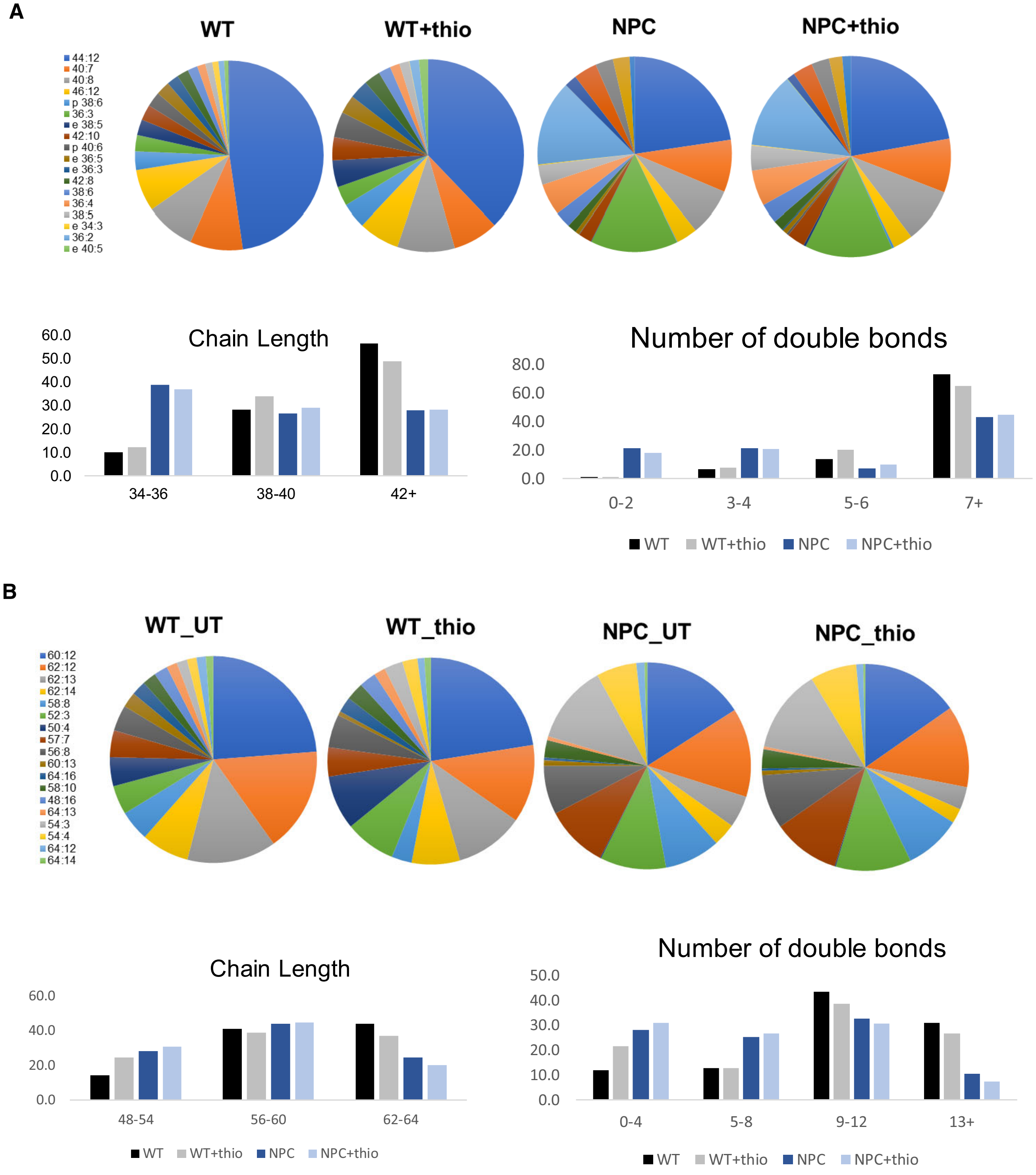

Figure EV5. Distribution of LBPA and sLBPA species in mouse liver extracts.

A, B The species of LBPA (A) and sLBPA (B) were quantified by LC-MS (as in Appendix Figure S1) in liver extracts of $Npc1^{-/-}$ and $Npc1^{+/+}$ mice, treated or not with thioperamide (as in Fig 6B and Appendix Figs S1 to S3), and are expressed as a percentage of the total. The colour code indicates the acyl chain composition of the various species listed in the captions, with the total number of carbon atoms of the 2 (LBPA) or 3 (sLBPA) acyl chains together, and the degree of insaturation. The prefix "e" indicates lipids with one 1-O-alkyl bond; prefix "p" indicates lipids with one 1-O-alkenyl bond. The bottom panels recapitulate the data after grouping lipid species by the number of carbon atoms in 2 (LBPA) or 3 (sLBPA) acyl chains (chain length) or the degree of insaturation (number of double bonds).

© 2019 The Authors
